# Colorectal cancer subtype identification from differential gene expression levels using minimalist deep learning

**DOI:** 10.1186/s13040-022-00295-w

**Published:** 2022-04-23

**Authors:** Shaochuan Li, Yuning Yang, Xin Wang, Jun Li, Jun Yu, Xiangtao Li, Ka-Chun Wong

**Affiliations:** 1 Department of Information Science and Technology, Northeast Normal University, Changchun, Jilin China; 2 Department of Surgery, Chinese University of Hong Kong, Hong Kong SAR, China; 3 Department of Infectious Diseases and Public Health, Jockey Club College of Veterinary Medicine and Life Sciences, and School of Data Science, City University of Hong Kong, Hong Kong SAR, China; 4 Institute of Digestive Disease and Department of Medicine and Therapeutics, State Key Laboratory of Digestive Disease, Li Ka Shing Institute of Health Sciences, The Chinese University of Hong Kong, Sha Tin, Hong Kong SAR, China; 5 School of Artificial Intelligence, Jilin University, Changchun, Jilin China; 6 Department of Computer Science, City University of Hong Kong, Hong Kong SAR, China

**Keywords:** DeepCSD, Cancer subtype identification, Differential gene expression

## Abstract

**Background:**

Cancer molecular subtyping plays a critical role in individualized patient treatment. In previous studies, high-throughput gene expression signature-based methods have been proposed to identify cancer subtypes. Unfortunately, the existing ones suffer from the curse of dimensionality, data sparsity, and computational deficiency.

**Methods:**

To address those problems, we propose a computational framework for colorectal cancer subtyping without any exploitation in model complexity and generality. A supervised learning framework based on deep learning (DeepCSD) is proposed to identify cancer subtypes. Specifically, based on the differentially expressed genes under cancer consensus molecular subtyping, we design a minimalist feed-forward neural network to capture the distinct molecular features in different cancer subtypes. To mitigate the overfitting phenomenon of deep learning as much as possible, *L*_1_ and *L*_2_ regularization and dropout layers are added.

**Results:**

For demonstrating the effectiveness of DeepCSD, we compared it with other methods including Random Forest (RF), Deep forest (gcForest), support vector machine (SVM), XGBoost, and DeepCC on eight independent colorectal cancer datasets. The results reflect that DeepCSD can achieve superior performance over other algorithms. In addition, gene ontology enrichment and pathology analysis are conducted to reveal novel insights into the cancer subtype identification and characterization mechanisms.

**Conclusions:**

DeepCSD considers all subtype-specific genes as input, which is pathologically necessary for its completeness. At the same time, DeepCSD shows remarkable robustness in handling cross-platform gene expression data, achieving similar performance on both training and test data without significant model overfitting or exploitation of model complexity.

**Supplementary Information:**

The online version contains supplementary material available at (10.1186/s13040-022-00295-w).

## Introduction

The gene expression-based consensus molecular subtyping has been demonstrated as a promising paradigm for patient stratification and therapy [1]. Molecular subtyping of cancer is an indispensable step toward individualized treatments while providing important biological insights into cancer heterogeneity [2]. However, traditional cancer diagnosis heavily relies on manual inspections and human clinician expertise [3, 4]. Moreover, the traditional diagnosis methods of cancer subtypes are too expensive that many patients give up treatment [5]. To address those problems, computational methods have been developed to diagnose cancer subtypes from molecular data. Unfortunately, since those molecular data are collected with the high dimensionality of the gene space and only a few cancer subtypes are available for different cancers, the sufficient data collection issues for cancer classification after reducing the dimensionality of gene space become particularly important. Meanwhile, the limited number of cancer subtypes can expose the computational methods vulnerable to model overfitting.

In the past, application-specific supervised cancer diagnosis methods have been developed to address the previously mentioned challenges; for instance, Yang et al. [6] constructed four models based on support vector machine (SVM) for predicting the metastasis of esophageal squamous cell carcinoma. Huang et al. [7] discussed the performance of the SVM-based model for predicting the response of cancer patients to drugs. Wang et al. [8] proposed an improved method based on random forest (RF) for lung cancer classification [9]. However, those methods usually suffer from realistic restrictions such as high dimensionality and computational scalability.

Recently, deep learning has been proposed to deal with those problems. It automatically generates informative features from low-dimensional features to discover the essential data representations [10]. Several supervised models based on deep learning have been employed to address cancer classification task successfully; for instance, Zeng et al. [11] applied DeepCues, a convolutional neural network, to classify seven major cancers based on DNA sequences. Islam and Poly [12] proposed a feedforward neural network to model breast cancer risk. Karabulut and Ibrikci [13] illustrated deep belief networks to address cancer classification problem and demonstrated impressive performance over SVM and RF [14, 15]. Sveen et al. [1] proposed a model called PDX (patient-derived xenograft) to focus on CMS-specific drug sensitivity. DeepCC, a cancer molecular subtype classification based on deep learning framework, has been developed for predicting consensus molecular subtypes based on high-dimensional gene expression profiles [2]. Unfortunately, most of the classifiers are subject to certain limitations. Firstly, the gene signature method only emphasizes the role of individual genes, while not fully considering the pathological impacts. Moreover, the overfitting phenomenon is recurring due to model complexity and data redundancy [10]. Therefore, in this study, we proposed DeepCSD to address those problems.

At the beginning, to emphasize the pathological importance of different genes in identifying cancer subtypes, differential gene expression analysis is computed to identify different subtype-specific genes in each pair of subtypes. Secondly, we proposed a novel deep learning framework for cancer-subtype diagnosis, named DeepCSD. To avoid overfitting to the maximum extent possible, the dropout layer, *L*_1_ and *L*_2_ regularization are added into the network architecture to enhance the robustness of DeepCSD. Finally, to demonstrate the performance, we collected eight independent datasets to train and validate DeepCSD. The experimental results reveal that DeepCSD can achieve competitive performance over the current state-of- the-art cancer-subtype diagnosis methods including Random Forest (RF), deep forest (gcForest), support vector machine (SVM), XGBoost, and DeepCC. In addition, we directly applied the DeepCSD model to independent TCGA data and can characterize differential gene expressions among diverse marker genes. Meanwhile, gene ontology enrichment, and KEGG pathology analysis are conducted to reveal novel insights into the cancer subtype identification and characterization mechanisms with validations.

## Methods

### Datasets

In this study, we collected eight independent colorectal cancer datasets [16] including GSE13067, GSE13294, GSE14333, GSE17536, GSE20916, GSE2109, GSE37892, and GSE39582 (Supplementary Table S1). All those datasets can be downloaded from the official repository of an international CRC subtyping consortium on Synapse (https://www.synapse.org/#!Synapse:syn2623706/wiki/) (downloaded on 1 Oct 2020).

### Gene expression analysis

Since the tumor microenvironment makes an important contribution to gene expression [1], we identify all differential genes with discriminative gene expression values among the cancer subtypes (namely, subtype-specific genes).

In this study, we focus on the well-established consensus molecular subtypes (CMS), i.e. CMS1-CMS4. Each subtype is characterized by its own unique feature: CMS1 (microsatellite instability immune), CMS2 (canonical), CMS3 (metabolic), and CMS4 (mesenchymal). The definitions can be found in [16]. Such high-dimensional data often brings difficulties in computational resources (i.e., computer memory). Therefore, the primary task is to select meaningful features from such high-dimensional gene expression data. Recognizing that there were four consensus molecular subtypes in this study, we combined these subtypes into six groups: CMS1 vs CMS2, CMS1 vs CMS3, CMS1 vs CMS4, CMS2 vs CMS3, CMS2 vs CMS4, and CMS3 vs CMS4. The subtype-specific genes are identified based on the fold-change and adjust *P* value (also called *Q* value). Mathematically, the fold-change can be defined as follows:

1 $$ {fold-change}=\frac{\overset{-}{A}}{\overset{-}{B}}  $$

while *A* and *B* denote the genes expression values of the same gene in different subtypes, $\overset {-}{A}$ is the average value of *A* and $\overset {-}{B}$ is the average value of *B*. In common, the *log*_2_ fold-change is widely used to normalize the range of fold-change. The *log*_2_ fold-change can be defined as follows:

2 $$ log_{2}\: \: {fold-change}=log_{2}{\overset{-}{A}}-log_{2}{\overset{-}{B}}  $$

However, the fold change has a drawback that the misclassification of differentially expressed genes with large differences may result in poor identification of changes in high expression levels.

To address it, the *P* value is implemented as an alternative to the rejection point and provided the minimum level at which the initial hypothesis will be rejected. The *P* value we used is adjusted by the Benjamini-Hochberg (BH) program [17], also called *Q* value. In this study, we take the *T-test* to compute every gene’s *P* value. More formally, the *T-test* is defined as follows:

3 $$ T=\frac{\overset{-}{A}-\overset{-}{B}}{\sqrt{S^{2}_{A}/n+S^{2}_{B}/n}}  $$

Then the significance *P* value is calculated according to the *T* distribution to measure the significance of this difference. Next, we took BH to obtain a *Q* value. More formally, the *Q* value in this study is defined as follows:

4 $$ Q=BH(P)  $$

After that, we calculated the fold-change and *Q* value for each CMS group. Then, the genes with ∣*log*_2_ fold-change ∣>1 and *Q* value <0.05 are retained and identified. Finally, we input those subtype-specific genes into deep learning and then construct our DeepCSD model.

### Deep learning for cancer subtype diagnosis (DeepCSD)

**Fig. 1:**
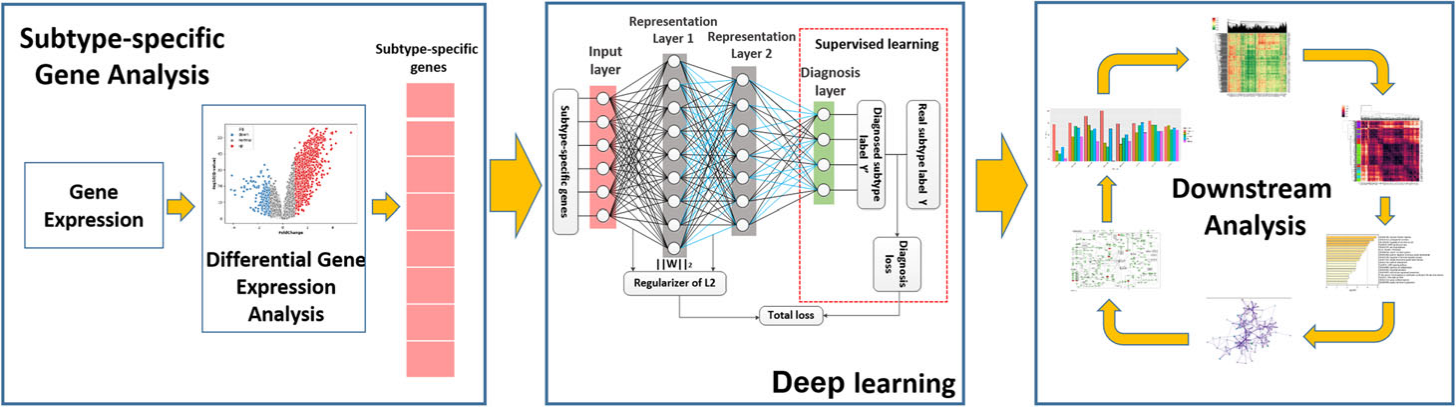
The framework of DeepCSD. DeepCSD consists of two representation layers to extract deep features, two dropout layers, and a output layer. DeepCSD has 768 neurons in the first representation layer, 128 in the second representation layer, and 4 in the final layer because molecular subtyping of colorectal cancer is a four-class classification problem in our study

Our deep neural network (DNN) is a feed-forward neural network assembled by a sequential layer-by-layer structure that realizes a series of functional transformations. Each layer is fed with the previous layer’s outputs as its inputs is to execute its transformation function. Specifically, every layer consists of multiple “neurons”. The input data will pass every layer by the “connections” with the weight parameters between “neurons”. The basic layers in DeepCSD are the representation layers and dropout layer (as shown in Fig. 1). DeepCSD features the sequential alternating representation layers with nonlinear activation functions (i.e. ReLU) and dropout layers, followed by representation layers with softmax activation functions for computing probability of each CMS (more detail can be seen in Fig. 1). Mathematically, it is recursively defined as follows:

5 $$ f^{l}(X)=\alpha_{l}(W_{l}R_{l-1}f^{l-1}(X)-\theta_{l})\: \: \:l=1,2,3  $$

while *X* is the model’s inputs, *f*^*l*−1^(*X*) is the (*l*−1)-th layer’s output, *θ*_*l*_ is the *l*-th layer’s threshold, and *α*_*l*_ is the *l*-th layer’s activate function. It is worth noticing that *R*_0_ does not exist. In other words, the dropout layer is not employed in DeepCSD’s first layer.

The natural method is to process the original inputs layer by layer to obtain “deep features”. In other words, every layer in the framework of deep learning is to extract deep features from the previous layer’s outputs. The more layers the DNN has, the deeper features the model can extract. In theory, the addition of layers and parameters can increase the model expressiveness for addressing complex learning tasks. However, the possibility of overfitting is also increased [10]. To reduce the influence of overfitting, *L*_2_ regularization and dropout layers are employed in our model. For the dropout layer, it is to abandon a part of neurons to mitigate the overfitting. The brief implementation step is defined as follows:

6 $$ R=Bernoulli(p)  $$

7 $$ f^{'}(x)=R*f(x)  $$

where the Bernoulli function is to randomly generate a binary mask vector with Bernoulli trial probability *p*, *f*(*x*) is the input of dropout layer, and $\phantom {\dot {i}\!}f^{'}(x)$ is the output [18].

After that, DeepCSD applies *L*_1_ and *L*_2_ regularization to representation layers 1 and 2 by adding a penalty term to the empirical loss. It is worth to be noticed that the output layer in DeepCSD does not employ such regularization.

### Training of the DeepCSD model

For such a multi-class classification task, we minimized the objective function of DeepCSD defined as follow:

8 $$ min\;\;loss=\sum_{i=0}^{n}\sum_{t=0}^{3}y_{i,t}ln(y_{i,t}^{'})+\frac{\lambda }{2}||W||_{1}+\frac{\lambda }{2}||W||_{2}  $$

while *n* is the number of samples, $y_{i,t}^{'}$ is the *t*-th neuron’s output of *i*-th sample, *λ* is the learning rate, $\frac {\lambda }{2}||W||_{1}$ is the term of *L*_1_,and $\frac {\lambda }{2}||W||_{2}$ is the term of *L*_2_. With one-hot label vector encoding, the objective function can be simplified as follow:

9 $$ {\displaystyle \begin{array}{c}\mathit{\min}\kern5.55pt loss=\sum \limits_{i=0}^n\mathit{\ln}\left({y}_{i,t}^{\ast}\right)+\frac{\lambda }{2}{\left\Vert W\right\Vert}_2\\ {} subject\kern0.34em to\kern5.55pt {y}_{i,t}=1,\sum \limits_{j=0}^3{y}_{i,j}=1\end{array}} $$

while $y_{i,t}^{*}$ indicates the output label index of the element 1 in one-hot vector for *i*-th sample.

The Adam algorithm has been chosen as our model’s optimizer [19]. The update rule is defined as follow:

10 $$ \Theta_{t}=\theta_{t-1}-\alpha \frac{m_{t}}{1-\beta_{1}^{t}}/(\frac{v_{t}}{1-\beta_{2}^{t}}+\varepsilon)  $$

while

11 $$ m_{t}=\beta_{1}m_{t-1}+(1-\beta_{1})g_{t}  $$

12 $$ v_{t}=\beta_{2}m_{t-1}+(1-\beta_{2})g_{t}^{2}  $$

13 $$ g_{t}=\bigtriangledown_{\Theta} f_{t}(\theta_{t-1})  $$

We can see that if we compute the parameter of *g*_*t*_, the Adam algorithm automatically updates the corresponding parameter. Next, we take the update process of *W*_3_ as a sample to explain the detail of our model update process. Mathematically, the output is defined as follow:

14 $$ y_{i,t}^{*}={softmax}(W_{3}R_{2}f^{2}(X_{i})-\Theta_{3})  $$

We focus on the parameter updating process of *W*_3_. The Adam algorithm makes some adjustment to *W*_3_ if the *g*_*t*_ is computed. The *g*_*t*_ for *W*_3_ is calculated as follow:

15 $$ g_{t}=\frac{\partial loss}{\partial W_{3}}  $$

while

16 $$ \frac{\partial loss}{\partial W_{3}}= \frac{\partial lny_{i,t}^{*}}{\partial y_{i,t}^{*}}\frac{\partial y_{i,t}^{*}}{\partial (W_{3}R_{2}f^{2}(x)-\Theta_{3})}\frac{\partial W_{3}R_{2}f^{2}(x)-\Theta_{3}}{\partial W_{3}}  $$

17 $$ \frac{\partial loss}{\partial W_{3}}= y_{i,t}^{*-1}\frac{\partial y_{i,t}^{*}}{\partial (W_{3}R_{2}f^{2}(x)-\Theta_{3})}R_{2}f^{2}(x)  $$

Next, we focus on partial derivative calculation of activation function for (*W*_3_*R*_2_*f*^2^(*x*)−*Θ*_3_). Firstly, the partial derivative calculation of softmax activation function for *k*-th neuron is calculated as follow:

18 $$ \frac{\partial y_{i,t}^{*}}{\partial k}= \frac{\frac{\partial e^{i,t}}{\partial k}\sum_{j=0}^{3}e^{i,j}-e^{i,t}\sum_{j=0}^{3}\frac{\partial e^{i.j}}{\partial k}}{(\sum_{j=0}^{3}e^{i,j})^{2}}  $$

It can result in two different outputs according to the value of *k*, i.e.

19 $$ \frac{\partial y_{i,t}^{*}}{\partial k}= \left\{ \begin{array}{ll} \frac{e^{i,t}\sum_{j=0}^{3}e^{i,j}-(e^{i,t})^{2}}{(\sum_{j=0}^{3}e^{i,j})^{2}}=y_{i,t}^{*}(1-y_{i,t}^{*}) & if(t=k)\\ \frac{e^{i,t}e^{i,k}}{(\sum_{j=0}^{3}e^{i,j})^{2}}=-y_{i,t}^{*}y_{i,k}^{*} & if(t\neq k) \end{array}\right.  $$

Therefore, we view *W*_3_ as (*w*_1_,*w*_2_,*w*_3_,*w*_4_)^*T*^ (expand matrix *W*_3_ by row). Then, the partial derivative calculation of activation function for (*W*_3_*R*_2_*f*^2^(*x*)−*Θ*_3_) is divided in two situations as follows:

20 $$ \frac{\partial y_{i,t}^{*}}{\partial (w_{k}R_{2}f^{2}(x)-\Theta_{3,k})}= \left\{ \begin{array}{ll} y_{i,t}^{*}(1-y_{i,t}^{*}) & if(k=t)\\ -y_{i,t}^{*}y_{i,k}^{*} & if(k\neq t) \end{array} \right.  $$

The final *g*_*t*_ is given by the following formula:

21 $$ \frac{\partial loss}{\partial w_{k}}= \left\{ \begin{array}{ll} (1-y_{i,t}^{*})R_{2}f^{2}(x)& if(k=t)\\ -y_{i,k}^{*}R_{2}f^{2}(x)& if(k\neq t) \end{array} \right.  $$

Combining those column vectors into a matrix in order, the *g*_*t*_ appeared. The $y_{i,t}^{*}$ is the neuron’s output that its position is consistent with the real label, which means the possibility of the real label. We can conclude that *w*_*k*_ will be strengthened if “ *k*=*t*”; it will be punished if “ *k*≠*t*”.

### Model parameters and running time

In this study, those eight molecular datasets are employed for experimental comparisons. For the DeepCSD, the parameters of Adam follows the default setting [20] and the minimum learning rate was set to 10^−5^. For SVM and RF, the parameter setting follows the default parameter values of Python Scikit-learn 0.21.2. For gcForest, we fixed this model to a multi-class mission with the literature default setting [22]. For XGBoost, *gamma*=0.1, *maxdepth*=12, *subsample*=0.7, *colsamplebytree*=0.7, *earlystoppingrounds*=15. For DeepCC, all parameter setting follows the reference [2]. Moreover, we also provide the running time of our model. DeepCSD is written in Python. We have relied on a server with CPU = Intel(R) Xeon(R) CPU E5-2620 v4 @ 2.10GHz, GPU = GTX1080 Ti. Meanwhile, we design our deep learning framework based on Keras. The versions of those software and packages are Python =3.7, Anaconda=3.7, Keras=2.1.0, Tensorflow=1.14.0. After running on those molecular datasets, the average running time is 457 seconds.

## Results

### Competing methods

To comprehensively demonstrate the DeepCSD performance, five state-of-the-art cancer-subtype diagnosis methods including Random Forest (RF), multi-Grained Cascade Forest (gcForest), support vector machine (SVM), XGBoost, and DeepCC are employed in this study. Firstly, two traditional frameworks were constructed based on RF and SVM. The fundamental idea of SVM is to find a hyperplane in a given sample space to distinguish samples with different classes [10]. Although the initial aim of SVM is to address the two-class task, Hsu and Lin [21] improved it to the multi-class task. RF is an ensemble learning algorithm in which component learner is a decision tree known for its random feature selection method. Then, deep forest (gcForest) framework is a deep forest ensemble to address classification task and the final output is generated by a vote of each tree [22]. XGBoost is a kind of boosting method based on the decision tree, which is a powerful machine learning method known for “regularized boosting”. DeepCC is a deep learning method as one of the comparative models with five hidden layers. Moreover, we also compared our DeepCSD with other deep learning methods (DeepCC) and deep forest (gcForest) to demonstrate the effectiveness of differential gene expression analysis in our model.

### Evaluation metrics

Four evaluation metrics have been measured in this study. For each dataset, according to the relationship between the real subtype and the diagnosis of DeepCSD, each diagnosis of DeepCSD was divided into true positive (TP), true negative (TN), false positive (FP), and false negative (FN). These four-measure metrics are defined as follow:

22 $$ {Sensitivity} = \frac {TP}{TP+FN}*100\%  $$

23 $$ {Specificity} = \frac {TN}{TN+FP}*100\%  $$

24 $$ {Precision} = \frac {TP}{TP+FP}*100\%  $$

25 $$ {Accuracy}=\frac{1}{n}\sum_{i=1}^{n}I(y_{i}=y_{i}^{*})*100\%  $$

which *y*_*i*_ is the sample’s true label, $y_{i}^{*}$ is the diagnosis of DeepCSD, *I*(*x*) is the indicator function, and *n* is the samples of each molecular data.

### Application to cancer gene expression data

**Fig. 2:**
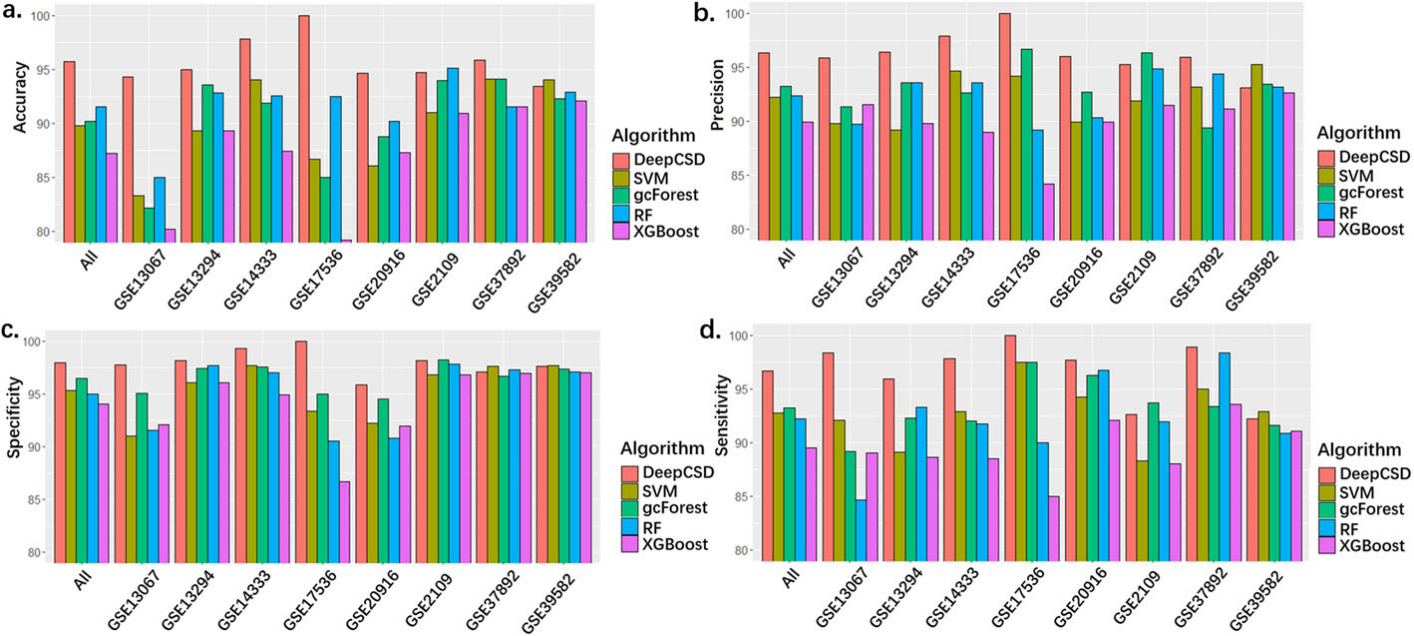
Performance comparisons among DeepCSD, SVM, gcForest, and RF on eight cancer molecular datasets. **a** accuracy (calculated by the mean of accuracy per class). **b** Precision (calculated by the mean of precision per class). **c** Specificity (calculated by the mean of specificity per class). **d** Sensitivity (calculated by the mean of sensitivity per class)
- Secondly, 1) gcForest and RF provide good performance since those methods are boosting methods. Its accuracy, specificity, and sensitivity are higher than 90% in 5 out of 8 datasets. The accuracy of RF is higher than gcForest on GSE20916, GSE14333, and GSE39582. However, the specificity of RF is lower than gcForest for those datasets. 2) gcForest, RF, SVM, XGBoost and DeepCSD can obtain better results than other compared algorithms on GSE39582 due to the influence of subtype-specific genes.
- Thirdly, the average accuracy of DeepCSD, gcForest, and RF are above 90% while SVM and XGBoost cannot provide such performance.
- Last but not least, we can find that its accuracy on GSE20916 and GSE2109 is not bad at all compared with other datasets. Moreover, we also computed the distance correlations [23] between those eight datasets as shown in Fig. 3. We can draw the conclusion that GSE20916 and GSE2109 have low correlation with other datasets, demonstrating DeepCSD’s robustness on different datasets with diverse gene expression characteristics.

To comprehensively demonstrate the performance of DeepCSD, all datasets (i.e. GSE13067, GSE13294, GSE37892, GSE39582, GSE2109, GSE14333, GSE17536, GSE20916) are adopted for model training with 10-fold cross-validation. We identified the differential genes as the inputs of SVM, GCF, RF, and XGBoost by subtype-specific gene analysis. The experimental results are summarized in Fig. 2. We can conclude several interesting phenomenons:

- Firstly, DeepCSD provides competitive performances in specificity, precision, sensitivity, and accuracy: 1) the accuracy of DeepCSD is higher than other classifiers in GSE13067, GSE17536, and GSE20916 by 5%, while the specificity and sensitivity is not less than 94%. 2) although the performance of DeepCSD is not the best in GSE39582 and GSE2109, the difference in accuracy is only lower than the champion by ∼1% while the sensitivity is higher than 92% and specificity is higher than 96%. 3) the average accuracy, precision, specificity, and sensitivity are higher than the second place by 2% ∼5%.

**Fig. 3:**
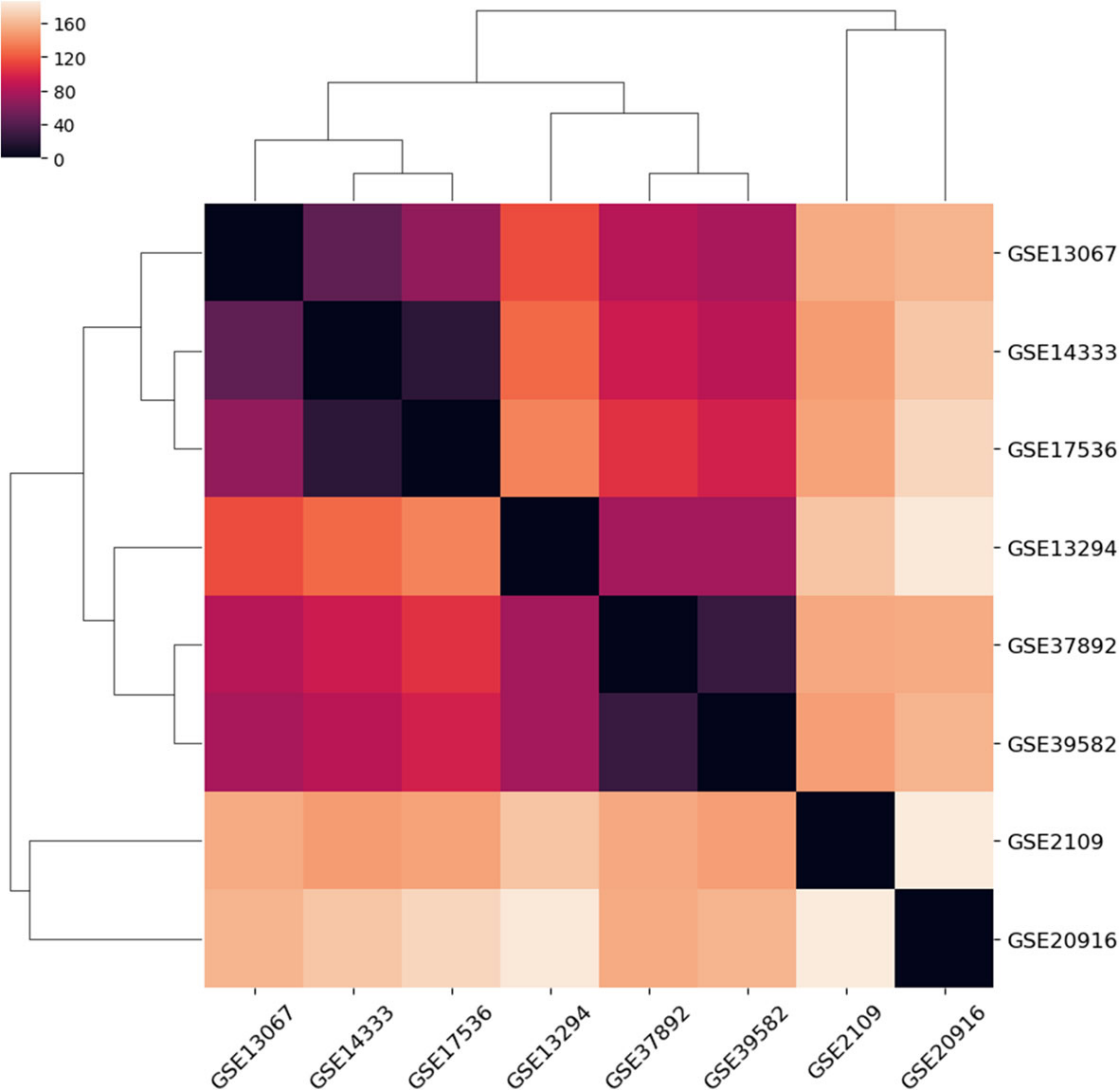
The distance correlation heatmap of multiple molecular datasets. From the figure, we can draw the conclusion that GSE20916 and GSE2109 have low correlations with other datasets; it demonstrates DeepCSD’s robustness on different datasets with diverse gene expression characteristics

### Subtype-specific gene analysis

To explore the influence of subtype-specific genes, GSE39582 was chosen as an example to reveal the subtype-specific genes’ contributions.

**Fig. 4:**
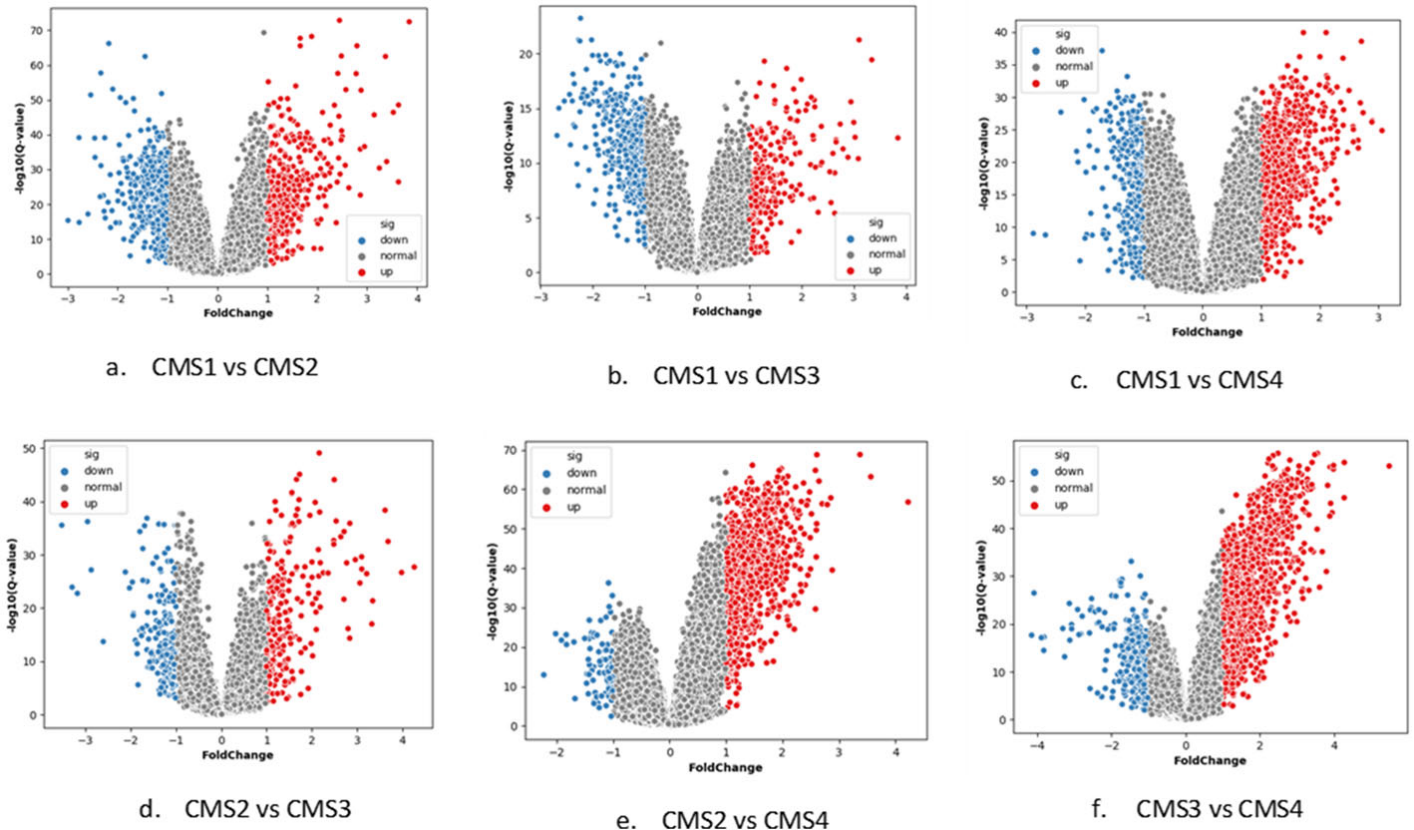
Differential gene expression visualization on GSE39582. **a**-**f** represents a comparison of each CMS group and each dot represents a gene: red represents up-regulated gene and blue represents down-regulated gene

In GSE39582, 2917 subtype-specific genes were extracted and visualized as red and blue dots in Fig. 4. From Fig. 4(a)-(f), we can draw some observations: firstly, all subtypes illustrate its distinctiveness since each CMS type has distinct up-regulated and down-regulated genes compared with others; secondly, it is a hard job to distinguish CMS2 due to the fact that the numbers of down-regulated gene are much lower than up-regulated genes compared with CMS3 and CMS4; last but not least, CMS4 is easy to diagnose given its distinctive characteristics in up-regulated genes.

**Fig. 5:**
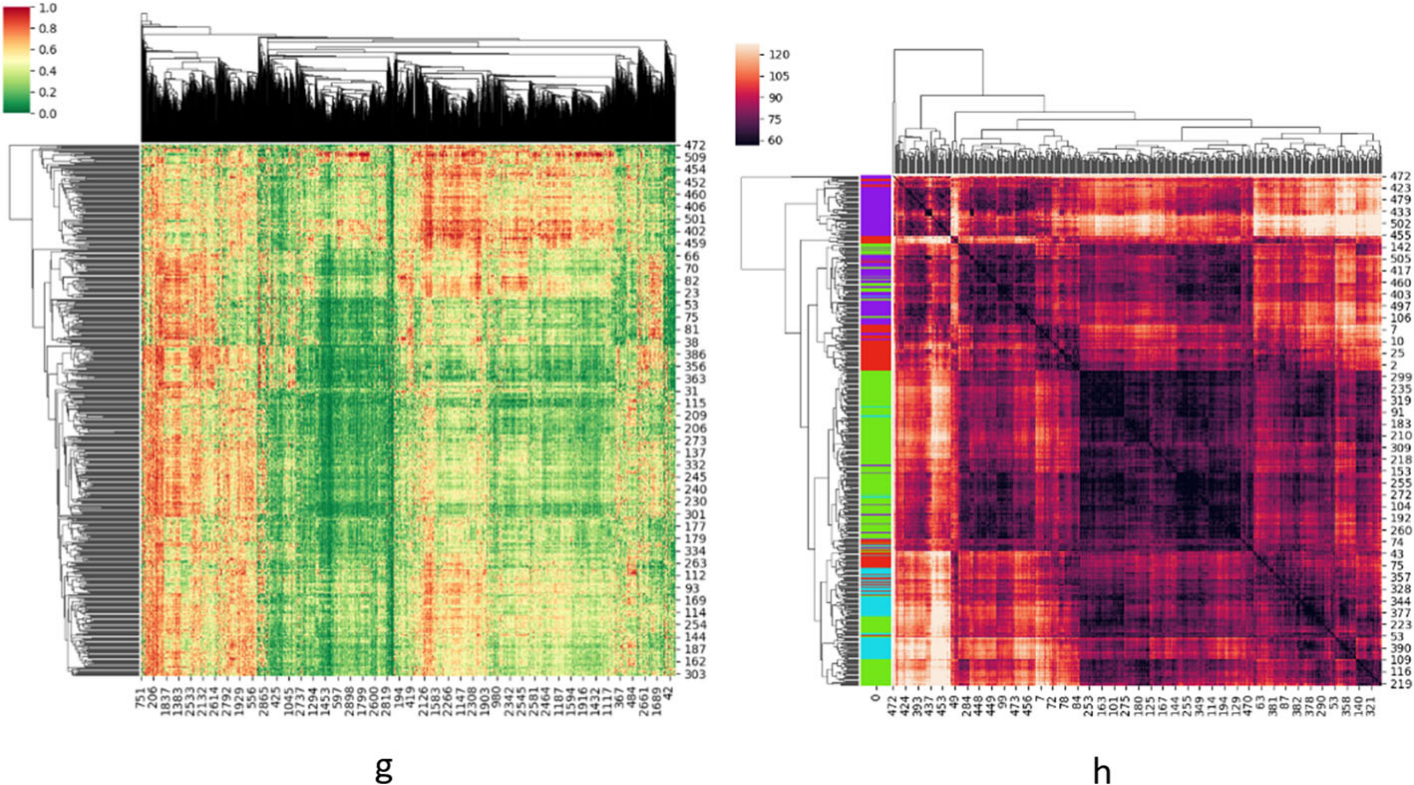
Gene Clustergram on GSE39582. **h** illustrates the difference between samples while **g** provides the genes’ correlation to each sample

To demonstrate the distinctiveness of those 2917 subtype-specific genes, we employed spectral clustering on all samples as depicted in Fig. 5. Figure 5(h) illustrates the difference between samples, while Fig. 5(g) provides the genes’ correlation to each sample. From Fig. 5, we can conclude that even with the differential gene analysis method, the distinguishability between the cancer patients remains very small.

To further demonstrate the subtype-specific genes’ performance, we compared our proposed DeepCSD with DeepCC. DeepCC combines samples’ gene expressions with public data to process gene set enrichment analysis (GSEA) to select some gene expressions which the authors called Functional Spectrum [2]. As mentioned above, DeepCC consists of five representation layers to extract “deep features” while DeepCSD only employed two layers as a minimalist approach. Therefore, it may not be a fair comparison for our proposed DeepCSD.

**Fig. 6:**
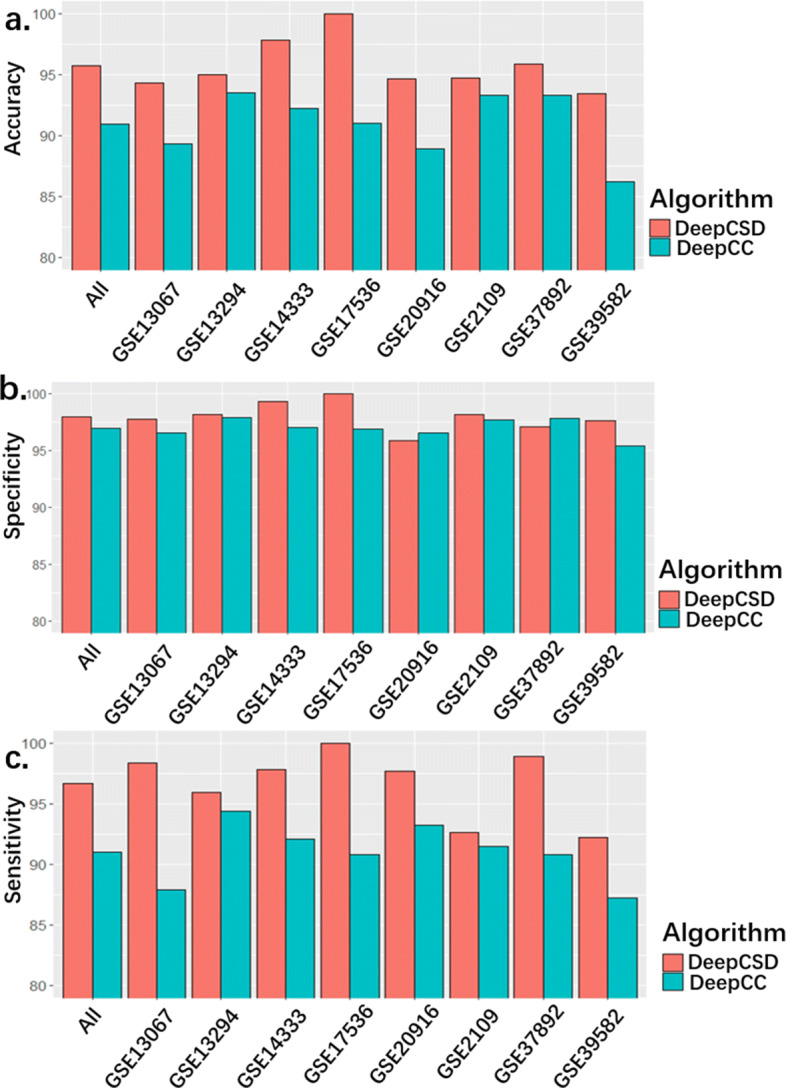
Performance comparison between DeepCSD and DeepCC. The vertical axis denotes the corresponding performance metric

**Fig. 7:**
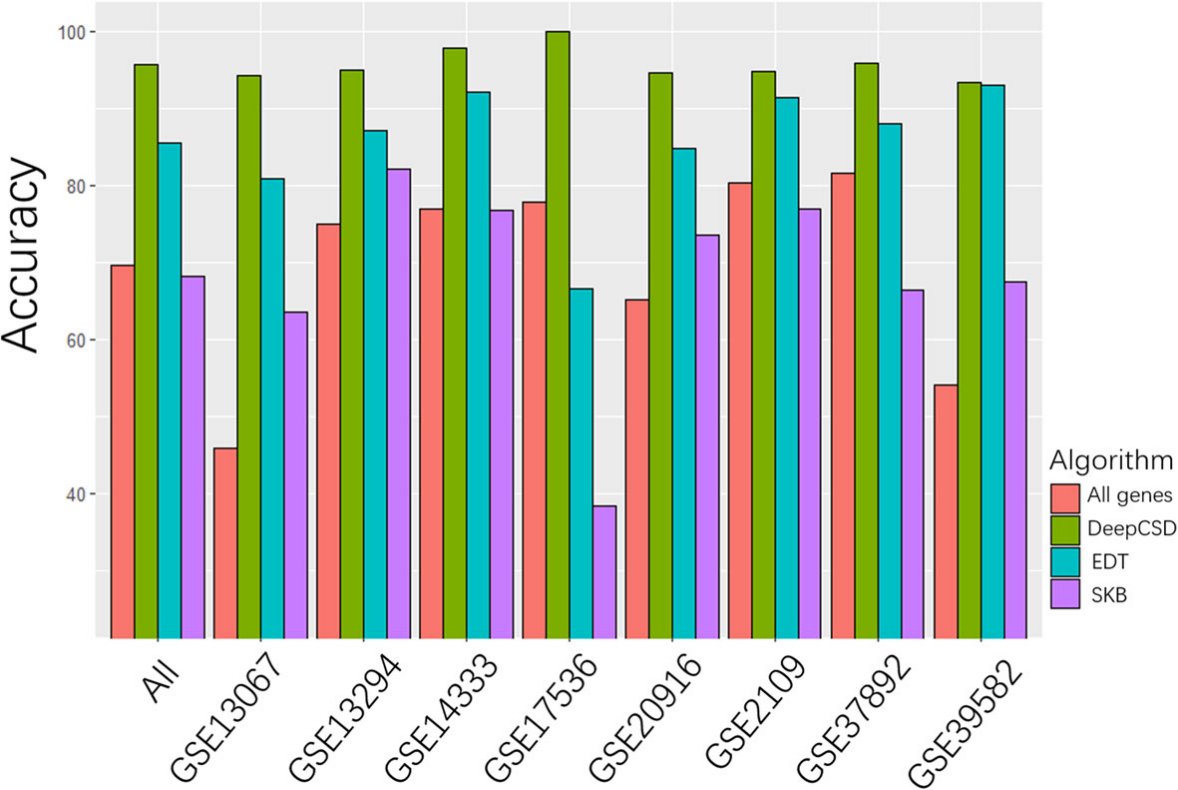
The performance comparisons of DeepCSD with two distinct feature selection methods including EDT and SKB

The experimental results are shown in Fig. 6. We can observe the followings: first of all, DeepCSD achieves higher performance than DeepCC in terms of accuracy, specificity, and sensitivity, while the average of accuracy and sensitivity is even higher than DeepCC by 5%. Meanwhile, although the specificity of DeepCSD is lower than DeepCC in GSE37892, the accuracy, and sensitivity of DeepCSD are higher than DeepCC. Given that, the subtype-specific gene provides a significant contribution to the diagnosis. To demonstrate the necessity of differential gene expression analysis, ensembles of decision trees (EDT) and select k best features (SKB) (supported by scikit-learn) were employed as feature selection methods for comparisons. Equally, the top 2000 genes selected by each method were selected as the input of our model. The results are summarized in Fig. 7.

It can be found that the performance of DeepCSD is higher than EDT and SKB under the same condition. Meanwhile, we also compared DeepCSD with and without differential genes. The results reveal that the differential genes analysis is very important in DeepCSD. Therefore, we can conclude that the subtype-specific differential gene expression analysis is an inevitable step towards cancer subtype diagnosis improvement.

### Parameter analysis

In DeepCSD, the dropout parameter is 0.5. It indicates that all inputs delivered from previous layers will be abandoned for half of the neurons. Traditionally, the parameter is chosen in the range from 0.2 to 0.5. However, to reduce the influence of overfitting to the utmost extent and to strengthen the generalization ability, dropout=0.5 is chosen in DeepCSD [18, 20].

**Fig. 8:**
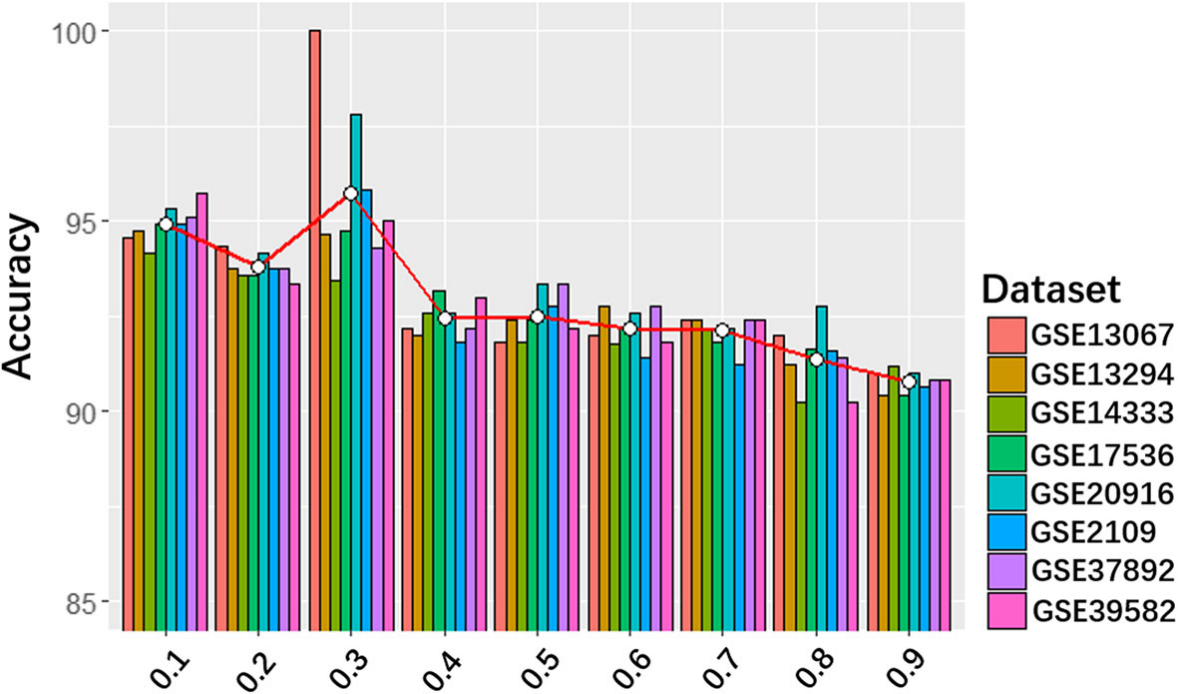
The comparison performance of different learning rates increasing from 0.1 to 0.9

The real challenge lies in the parameter setting of the regularizer. It is well known that the regularizer of *L*_1_ and *L*_2_ can strengthen the important features and reduce the weights of redundant features. Therefore, if the parameter is too large, the learning ability of DeepCSD will be limited. On the opposite, if the parameter is too small, the phenomenon of overfitting may not be prevented. To find a suitable parameter to strike a balance, we trained DeepCSD with the learning rate ranging from 0.1 to 0.9. The analysis is shown in Fig. 8.

We can summarize some conclusions from Fig. 8. In the first, the numerical variation of the average accuracy is not very large when the learning rate is in the range from 0.1 to 0.3. Secondly, with the increase in the learning rate, the average curve is falling. That is in line with the expected impact of the regularizer. More interestingly, the average accuracy is still higher than 90% even at learning rate=0.9. Finally, it can be observed that DeepCSD has the best performance at the learning rate =0.3. Therefore, we choose the learning rate as 0.3 in our study. Moreover, we also compared DeepCSD with and without regularization as shown in Supplementary Fig. S1. From this figure, the performance of DeepCSD with regularization is higher than DeepCSD without regularization.

## Case study

### The performance of DeepCSD on TCGA

To further demonstrate the performance of DeepCSD, we applied it to annotate subtypes on an independent colorectal cancer dataset from The Cancer Genome Atlas (TCGA) [16, 24]. The TCGA expression data can be downloaded from (https://www.synapse.org/#!Synapse:syn2623706/wiki/). The TCGA dataset has 512 patients with explicit CMS labels in total. Meanwhile, each patient sample in TCGA has 20293 features. In the first, the TCGA dataset was split randomly into a training set and test set with the 9:1 ratio. Next, we trained DeepCSD on a training set with 10-fold cross-validation and took the corresponding test set to demonstrate the performance of DeepCSD. To identify the differential genes, we applied the subtype-specific differential gene expression analysis, resulting in 4881 genes for such TCGA data. From Supplementary Fig. S3, we can find that CMS4 also illustrated its distinctive difference compared with other groups. Moreover, the TCGA dataset shown its unique characterization from the previous molecular data. Intuitively, DeepCSD was trained on each training set (n=460) and then applied it to the diagnosis of the independent test set (n=52). The experimental results are tabulated in SupplementaryTables S2 and S3.

From Supplementary Table S2, we can conclude that the performance of the test set is no worse than that of the training set, indicating that the impact from the overfitting phenomenon of DeepCSD may be minimized. Supplementary Table S3 explicitly lists the performance of DeepCSD on TCGA: 1), 2 out of 19 CMS2 was diagnosed to other subtypes since the corresponding number of up- and down-regulated genes is low as we discussed in Subtype-specific gene analysis section; 2), the sensitivity of CMS3 and CMS4 are 100% since a large number of up- and down-regulated genes are available for diagnosis.

### Biological interpretability from DeepCSD

In the previous section, we have demonstrated the competitive performance of DeepCSD based on subtype-specific genes. In this section, we investigated the biological significance of the subtype-specific genes.

**Fig. 9:**
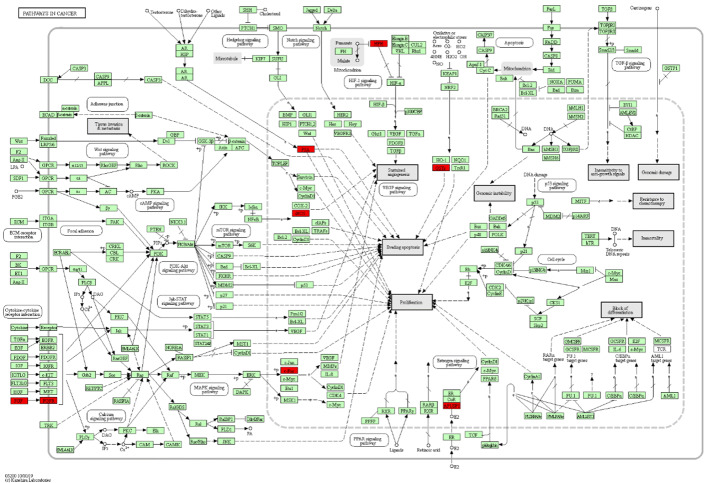
The metabolic pathways (hsa01100) is provided by KEGG database: the genes marked “red” are the subtype-specific genes in this pathway

Firstly, we identified the top 100 genes with the largest weights in each of the first 10 neurons in the representation layer 1 (the detail of those selected genes can be found in Supplemental Data). After that, we conduct gene ontology (GO) enrichment analysis based on Metascape [25] to analyze those selected genes. We input those selected genes into Metascape and then collect the GO enrichment in the first 10 neurons in the representation layer 1 of DeepCSD trained on the TCGA dataset. SupplementaryFig. S4 summarizes the results of GO enrichment analysis. In those figures, the top category is the enriched biological process ordered by *p*-values. After that, we randomly took the 9-th neuron as example; for instance, the top three enriched GO biological processes in 9-th neuron are mucosal immune response (GO:0002385), prostaglandin secretion (GO:0032310), and regulation of secretion by cell (GO:1903530). Meanwhile, as depicted in Fig. S2, the Cytoscape is employed to visualize a subset of enriched terms and present a network connected by edges to illustrate the relationship between terms. From the figure, each node denotes an enriched term and is colored first by its cluster ID as shown in the first figure of Fig. 9. After that, the *p*-values of the enriched term are clustered in the second figure of Fig. 9. Furthermore, some molecular pathways were provided by the enrichment analysis. For example, MAPR signaling pathway (hsa04010), PID HIF1 TFPATHWAY (M255, a kind of canonical pathways), cAMP signaling pathway (hsa04024), and Pancreatic secretion (hsa04972).

In addition, to further analyze the molecular pathways behind the layer of DeepCSD, those selected genes were taken to the molecular pathway analysis with KEGG (https://www.kegg.jp). As shown in Fig. 9, we observe that 7 subtype-specific genes of the 9-th neurons can be found in the PATHWAYS IN CANCER (hsa05200). That provide evidences that our subtype-specific genes make an extinguished contribution to diagnosis.

## Discussion and conclusions

Given the central importance of colorectal cancer subtyping, the consensus molecular subtype classification is always desirable for patient stratification. Specifically, the most important component to the consensus molecular subtyping is that the underlying classifier can diagnose every subtype correctly. In this study, we proposed a deep learning framework based on differential gene expression analysis to diagnose cancer subtypes.

Based on the results, we observe that DeepCSD can achieve better colorectal cancer subtype identification than RF, SVM, gcForest, XGBoost, and DeepCC. We also highlighted the significance of the differential gene expression analysis compared with other possible algorithms as shown in Fig. 7. Obviously, we can observe that the differential gene expression analysis is necessary and important for cancer subtype diagnosis. In particular, we conceive that DeepCSD can bring us the following performance advantages: 1) Biological interpretability: DeepCSD takes all of the subtype-specific genes into account as inputs, which are pathologically necessary for its completeness. 2) Robustness: DeepCSD demonstrated its remarkable robustness in processing cross-platform gene expression data. 3) Generalization ability: DeepCSD achieves similar performance on both training and test data, suggesting that it may not suffer from severe model overfitting or model complexity exploitation..

In the future, we are optimistic that our minimalist approach can demonstrate its own value and applicability to cancer genomics in the face of high-throughput medical data.

## Supplementary Information

**Additional file 1** Supplementary figures and tables.

## Data Availability

In this study, we collected eight independent colorectal cancer datasets [16] including GSE13067, GSE13294, GSE14333, GSE17536, GSE20916, GSE2109, GSE37892, and GSE39582 (Supplementary Table S1). All those datasets can be downloaded from the official repository of an international CRC subtyping consortium on Synapse (https://www.synapse.org/#!Synapse:syn2623706/wiki/) (downloaded on 1 Oct 2020)

## Abbreviations

DeepCSD: A supervised learning framework based on deep learning
RF: Random Forest
gcForest: Deep forest
SVM: support vector machine
DeepCC: a cancer molecular subtype classification based on deep learning framework
TCGA: The Cancer Genome Atlas
KEGG: Kyoto Encyclopedia of Genes and Genomes
CMS: consensus molecular subtypes
DNN: deep neural network
TP: true positive
TN: true negative
FP: false positive
FN: false negative
GSEA: gene set enrichment analysis
EDT: ensembles of decision trees
SKB: select k best features
