# Supplementary material for: Colorectal cancer subtype identification from differential gene expression levels using minimalist deep learning
Source: BioData Min. 2022 Apr 23;15:12. doi: 10.1186/s13040-022-00295-w (PMC9034628; doi:10.1186/s13040-022-00295-w)
Supplement: Supplementary file 1 — Additional file 1 Supplementary figures and tables. [file 13040_2022_295_MOESM1_ESM.pdf]

Table S1. Summary of CRC data sets

Dataset	Platform	Tissue	Total	with CMS*	Source	Number of genes
GSE13067	Affymetrix HG133plus2	Fresh frozen	74	67	CRCSC	54675
GSE13294	Affymetrix HG133plus2	Fresh frozen	155	140	CRCSC	54675
GSE37892	Affymetrix HG133plus2	Fresh frozen	130	118	CRCSC	54675
GSE39582	Affymetrix HG133plus2	Fresh frozen	566	519	CRCSC	54675
GSE2109	Affymetrix HG133plus2	Fresh frozen	293	266	CRCSC	54675
GSE14333	Affymetrix HG133plus2	Fresh frozen	290	135	CRCSC	54675
GSE17536	Affymetrix HG133plus2	Fresh frozen	177	38	CRCSC	54675
GSE20916	Affymetrix HG133plus2	Fresh frozen	145	71	CRCSC	54675

Table S2. The performance of DeepCSD on TCGA

	training set	test set
ACCURACY	93.04	94.23
precision	92.59	92.97
specificity	97.48	98.19
sensitivity	91.91	94.49

Table S3. The detail of DeepCSD on TCGA test set

	CMS1	CMS2	CMS3	CMS4
TP	7	19	7	16
TN	44	31	43	35
FN	1	2	0	0
FP	0	0	2	1
precision	1	1	0.777778	0.941176
specificity	1	1	0.955556	0.972222
sensitivity	0.875	0.904762	1	1

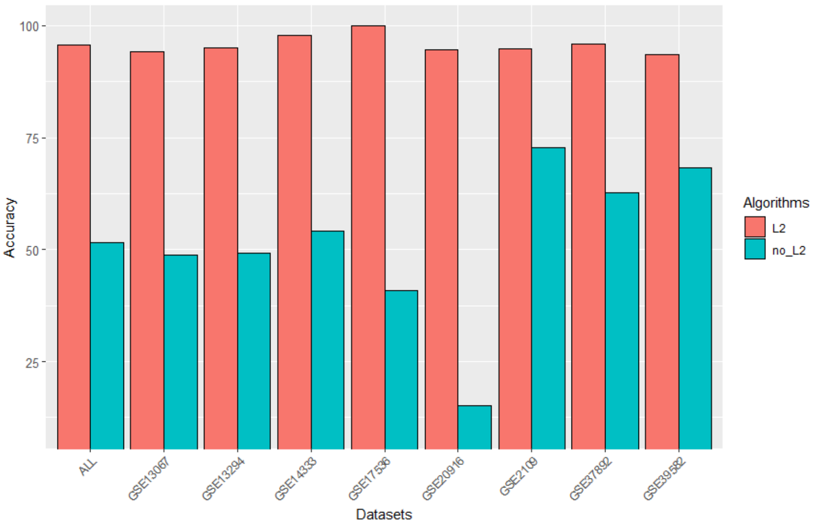

Figure S1. Performance comparison of DeepCSD with and without L_2 regularization.

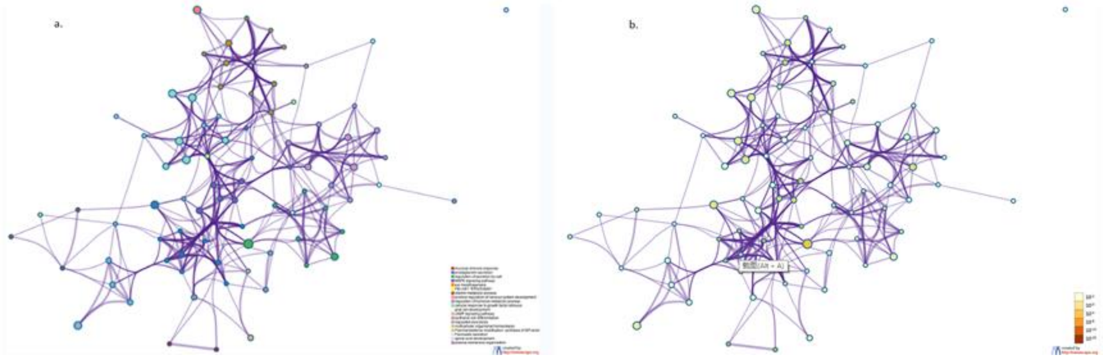

Figure S2. Similarity network of enriched terms about the top 100 subtype-genes in 9-th neuron with similarity > 0.3. (Left) network of enrichment terms,colored by cluster ID, where nodes that with the same cluster ID are typically close to each other. (Right) network of enrichment terms, colored by p-value, where the terms with many genes tend to have a more significant p-value.

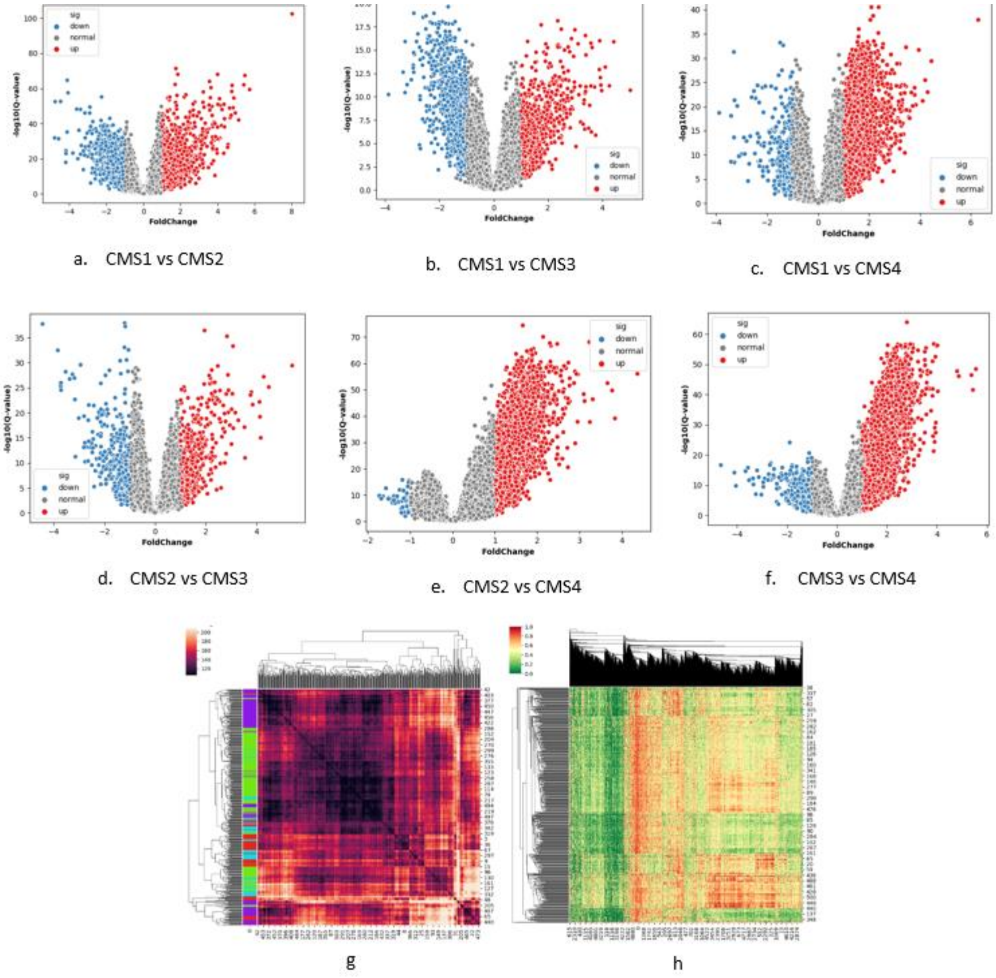

Figure S3. TCGA gene expression analysis. (a)-(f) represents a comparison of each CMS group and each dot represents a gene: red represents up-regulated gene and blue represents down-regulated gene. (h) illustrates the difference between samples while (g) provides the genes' correlation to each sample.

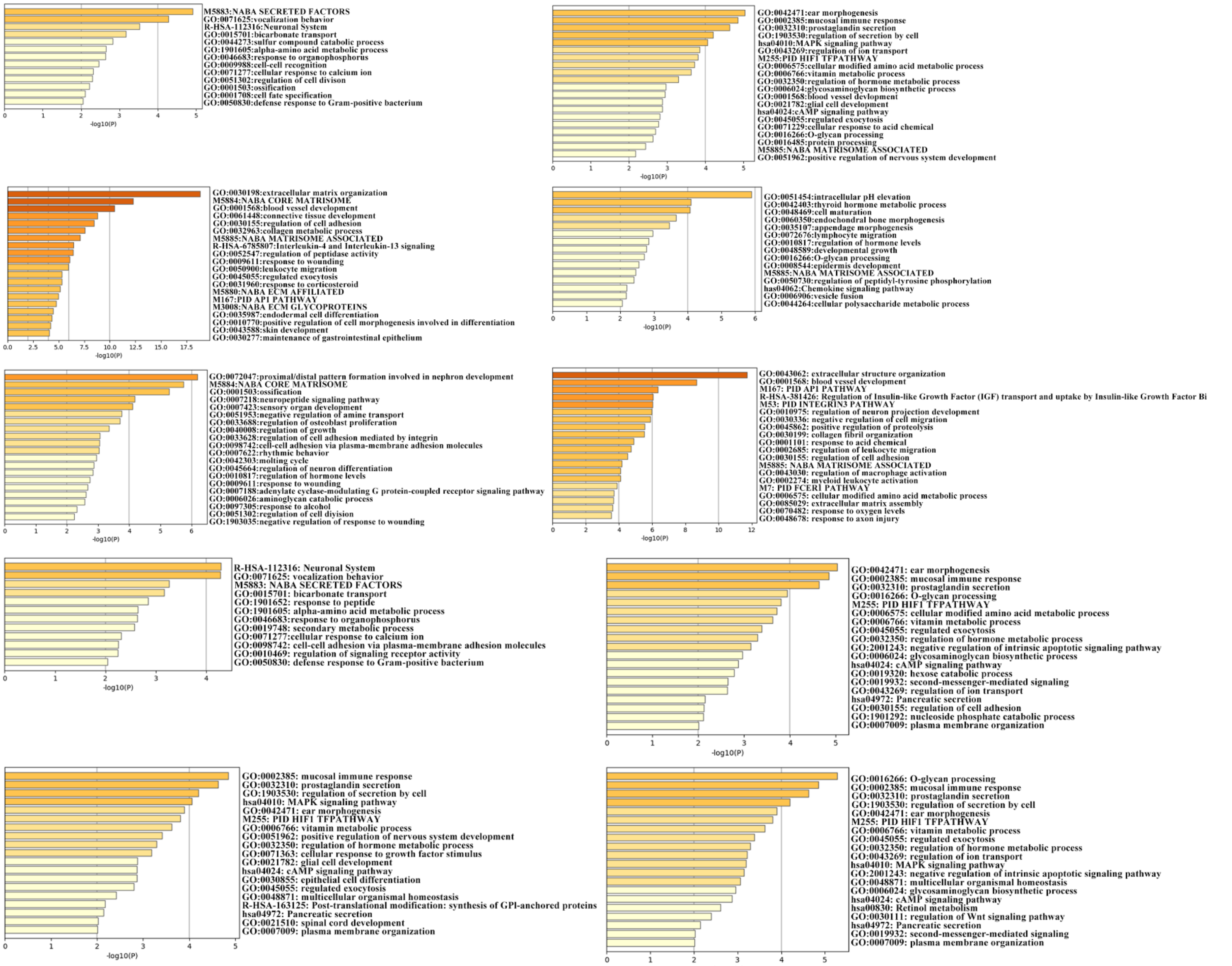

Figure S4. GO enrichment analysis of TCGA for the first ten neurons. Bar charts are plotted for the highly enriched GO terms across input gene lists as sorted and colored by p-values.
